# The Impact of *Fusarium* Mycotoxins on Human and Animal Host Susceptibility to Infectious Diseases

**DOI:** 10.3390/toxins6020430

**Published:** 2014-01-28

**Authors:** Gunther Antonissen, An Martel, Frank Pasmans, Richard Ducatelle, Elin Verbrugghe, Virginie Vandenbroucke, Shaoji Li, Freddy Haesebrouck, Filip Van Immerseel, Siska Croubels

**Affiliations:** 1 Department of Pharmacology, Toxicology and Biochemistry, Faculty of Veterinary Medicine, Ghent University, Salisburylaan 133, 9820 Merelbeke, Belgium; 2 Department of Pathology, Bacteriology and Avian Diseases, Faculty of Veterinary Medicine, Ghent University, Salisburylaan 133, 9820 Merelbeke, Belgium; E-Mails: An.Martel@UGent.be (A.M.); Frank.Pasmans@UGent.be (F.P.); Richard.Ducatelle@UGent.be (R.D.); Elin.Verbrugghe@UGent.be (E.V.); Shaoji.Li@UGent.be (S.L.); Freddy.Haesebrouck@UGent.be (F.H.); Filip.VanImmerseel@UGent.be (F.V.I.); 3 Animal Health Care Flanders, Industrielaan 29, 8820 Torhout, Belgium; E-Mail: Virginie.Vandenbroucke@dgz.be

**Keywords:** deoxynivalenol, fumonisin, *Fusarium* mycotoxins, human, infectious diseases, mouse, pig, poultry, T-2 toxin, zearalenone

## Abstract

Contamination of food and feed with mycotoxins is a worldwide problem. At present, acute mycotoxicosis caused by high doses is rare in humans and animals. Ingestion of low to moderate amounts of *Fusarium* mycotoxins is common and generally does not result in obvious intoxication. However, these low amounts may impair intestinal health, immune function and/or pathogen fitness, resulting in altered host pathogen interactions and thus a different outcome of infection. This review summarizes the current state of knowledge about the impact of *Fusarium* mycotoxin exposure on human and animal host susceptibility to infectious diseases. On the one hand, exposure to deoxynivalenol and other *Fusarium* mycotoxins generally exacerbates infections with parasites, bacteria and viruses across a wide range of animal host species. Well-known examples include coccidiosis in poultry, salmonellosis in pigs and mice, colibacillosis in pigs, necrotic enteritis in poultry, enteric septicemia of catfish, swine respiratory disease, aspergillosis in poultry and rabbits, reovirus infection in mice and Porcine Reproductive and Respiratory Syndrome Virus infection in pigs. However, on the other hand, T-2 toxin has been shown to markedly decrease the colonization capacity of *Salmonella* in the pig intestine. Although the impact of the exposure of humans to *Fusarium* toxins on infectious diseases is less well known, extrapolation from animal models suggests possible exacerbation of, for instance, colibacillosis and salmonellosis in humans, as well.

## 1. Introduction

Mycotoxins are toxic fungal metabolites that can contaminate a wide array of food and feed [1]. Mycotoxin-producing fungi can be classified into either field or storage fungi. Field fungi, such as the *Fusarium* species, produce mycotoxins on the crops in the field, whereas storage fungi, such as the *Aspergillus* and *Penicillium* species, produce mycotoxins on the crops after harvesting [2]. *Fusarium* fungi have traditionally been associated with temperate climatic conditions, since they require somewhat lower temperature for growth and mycotoxin production than, for example, the *Aspergillus* species [3]. The most toxicologically important *Fusarium* mycotoxins are trichothecenes (including deoxynivalenol (DON) and T-2 toxin (T-2)), zearalenone (ZEN) and fumonisin B1 (FB1).

*Fusarium* mycotoxins are capable of inducing both acute and chronic toxic effects. These effects are dependent on the mycotoxin type, the level and duration of exposure, the animal species that is exposed and the age of the animal [4]. Intake of high doses of mycotoxins may lead to acute mycotoxicoses, which are characterized by well-described clinical signs [5,6]. Exposure of pigs to high concentrations of DON causes abdominal distress, malaise, diarrhea, emesis and even shock or death. Exposure of pigs to fumonisins can lead to pulmonary edema due to cardiac insufficiency. In horses fumonisins can cause equine leukoencephalomalacia (ELEM) and target the brain [7]. Since these high contamination levels are rare in modern agricultural practice [8], this review will not discuss extensively their effect on animal or human health. Indeed, although the results of a global survey indicate that the *Fusarium* mycotoxins DON, fumonisins, and ZEN respectively contaminated 55%, 54% and 36% of feed and feed ingredients in the period 2004–2011, the majority of samples was found to comply with even the most stringent European Union regulations or recommendations on the maximal tolerable concentration (Table A3) [8]. Therefore, this review will focus on the effect of low to moderate doses of the major *Fusarium* mycotoxins.

**Figure 1:**
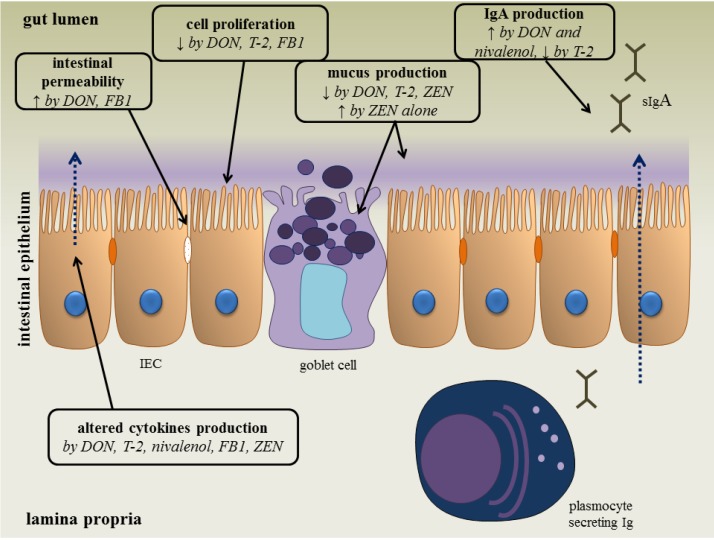
The effect of *Fusarium* mycotoxins on the intestinal epithelium. A variety of *Fusarium* mycotoxins alter the different intestinal defense mechanisms including epithelial integrity, cell proliferation, mucus layer, immunoglobulins (Ig) and cytokine production. (IEC: intestinal epithelial cell) (based on [9]).

Following oral intake of low to moderate amounts of these mycotoxins, the gastro-intestinal epithelial cell layer will be exposed first [9]. The intestinal mucosa acts as a barrier, preventing the entry of foreign antigens including food proteins, xenobiotics (such as drugs and toxins), commensal microbiota and pathogens into the underlying tissues [9,10]. The mucosal immunity, which consists of an innate and adaptive immune system, can be affected by *Fusarium* mycotoxins (Figure 1) [9,10]. An important component of the innate immune system are the intestinal epithelial cells, which are interconnected by tight junctions, and covered with mucus, produced by goblet cells [11]. By measuring the transepithelial electrical resistance (TEER), several *in vitro* and *ex vivo* studies indicate that DON and FB1 are able to increase the permeability of the intestinal epithelial layer of human, porcine and avian origin [12,13,14]. Also the viability and proliferation of animal and human intestinal epithelial cells can be negatively affected by *Fusarium* mycotoxins [9,15,16,17,18,19,20]. Their effect on mucus production is variable: co-exposure of low doses of DON, T-2 and ZEN reduces the number of goblet cells in pigs [21], but ZEN given alone at higher doses increases the activity of goblet cells [22]. Several mycotoxins are also able to modulate the production of cytokines *in vitro* and *in vivo* [9,23]. For example, DON increases the expression of TGF-β and IFN-γ in mice and fumonisins decrease the expression of IL-8 in an intestinal porcine epithelial cell line (IPEC-1) [9].

*Fusarium* mycotoxins can cross the intestinal epithelium and reach the systemic compartment [20,24], affecting the immune system. Exposure to these toxins can either result in immunostimulatory or immunosuppressive effects depending on the age of the host and exposure dose and duration [20,25]. Mycotoxin-induced immunomodulation may affect innate and adaptive immunity by an impaired function of macrophages and neutrophils, a decreased T- and B-lymphocyte activity and antibody production [23,25,26]. In addition to the effect of *Fusarium* mycotoxins on the animal or human host, these mycotoxins may alter the metabolism of the pathogen, which may alter the outcome of the infectious disease [27,28].

A wealth of research papers clearly indicate a negative influence of *Fusarium* mycotoxins on the intestinal function and immune system. Since the intestinal tract is also a major portal of entry to many enteric pathogens and their toxins, mycotoxin exposure could increase the animal susceptibility to these pathogens. Furthermore, mycotoxin-induced immunosuppression may also result in decreased animal or human host resistance to infectious diseases.

This review attempts to summarize the impact of *Fusarium* mycotoxin exposure on the animal and human host susceptibility to infectious diseases. More specifically, the effect of *Fusarium* mycotoxins on enteric, systemic and respiratory infectious diseases in livestock animals and animal models for human diseases are highlighted.

## 2. Effect of *Fusarium* Toxins on Parasitic Diseases

### Coccidiosis

Intestinal protozoa, including the coccidia (*Eimeria*, *Isospora*, *Cryptosporidium* and *Sarcosporidia*) and flagellates, are important infectious agents. Coccidiosis in poultry generally refers to the disease caused by the *Eimeria* species, and is still considered one of the most important enteric diseases affecting performance. These obligate intracellular parasites have an oral-fecal life cycle with developmental stages alternating between the external environment and the host [29].

Seven species of *Eimeria* (*E*. *acervulina*, *E*. *brunetti*. *E*. *maxima*, *E*. *mitis*, *E*. *necatrix*, *E*. *praecox* and *E*. *tenella*) are found in chickens [29]. The physical and biological characteristics, pathogenicity and immunogenicity depend on the species. Immunity to *Eimeria* is complex, multifactorial and influenced by both host and parasite [30].

Cell-mediated immunity, mainly evoked by the intraepithelial lymphocytes (IEL) and lymphocytes of the lamina propria, is the major protective immune component against avian coccidiosis [31,32]. The CD4^+^ T-lymphocytes, IEL and macrophages are involved in the response against primary exposure to *Eimeria* [31], while CD8^+^ T-lymphocytes and IFN-γ are important in the protective immune response against *Eimeria* infection [33]. Girgis *et al*. [34,35] showed a negative impact of diets naturally contaminated with *Fusarium* mycotoxins on the cell-mediated immune response against coccidiosis in broilers (Table A2). Following primary infection of broilers with *Eimeria*, *Fusarium* mycotoxins decreased the percentage of CD4^+^ and CD8^+^ T-cells in the jejunal mucosa [35]. In addition, feeding on a mycotoxin-contaminated diet lowered the blood levels of CD8^+^ T-cells and monocytes, which could suggest an increased recruitment at the intestinal site of coccidial infection or a delayed replication necessary to replenish these subsets in the circulation [34,35]. Additionally, feeding on a *Fusarium* mycotoxin-contaminated diet increased IFN-γ gene expression in the cecal tonsils of *Eimeria-*challenged birds, however, without being linked to the apparent resistance to coccidial infection in terms of changes in oocyst yield [34]. The cecal tonsils constitute a lymphoid tissue in the cecum belonging to the gut-associated lymphoid tissue (GALT). Resistance to *Eimeria* infection is related to the expression of a set of interleukins rather than only IFN-γ and the up-regulation of the gene may not necessarily be associated with functional secretion [34]. Furthermore, it was shown that moderate levels of *Fusarium* mycotoxins negatively affect intestinal morphology and interfere with intestinal recovery from an enteric coccidial infection, indicated by a lower villus height and apparent villus area (Table A2) [36]. Although Girgis *et al*. [34,35] demonstrated that *Fusarium* mycotoxins impair the *Eimeria-*induced immune response, no effect was seen on fecal oocyst counts. Similarly, Békési *et al*. [37] showed no impact of a T-2 and ZEN-contaminated diet on *Cryptosporidium baileyi* oocyst excretion in broilers.

Research investigating the influence of mycotoxins on the animal susceptibility to infectious diseases focuses mainly on exposure to single major mycotoxins. Limited information about the impact of mycotoxin co-occurrence and plant metabolites of mycotoxins on this interaction is available. Nevertheless, Girgis *et al*. [34,35] showed that the combination of DON, 15-acetylDON (15-AcDON), ZEN and fumonisins alters the *Eimeria*-induced immune response. Interestingly, mycotoxin contamination of broiler feed may reduce the efficacy of the anti-coccidial treatment with lasalocid [38].

To conclude, *Fusarium* mycotoxins negatively affect the innate and adaptive cellular immune response against *Eimeria*, though without changing the oocyst yield. Further data of clinical coccidiosis lesion scoring is still needed in order to evaluate the effect of *Fusarium* mycotoxins on the severity of the disease.

## 3. Effect of *Fusarium* Toxins on Bacterial Diseases

### 3.1. Salmonellosis

Salmonellosis is an infection with the Gram-negative *Salmonella* bacterium, a facultative anaerobic, facultative intracellular microorganism of the *Enterobacteriaceae* family. The host—*Salmonella* interaction is complex, with a broad array of mechanisms used by the bacteria to overcome host defenses. Two important disease manifestations are differentiated, *i*.*e*., gastroenteritis and enteric fever, caused by nontyphoidal and typhoidal *Salmonella* serovars, respectively [39].

Nontyphoidal *Salmonella* strains, such as *Salmonella* serovar Typhimurium and *Salmonella* serovar Enteritidis strains, infect a wide range of animal hosts, including pigs and poultry, without causing clinical symptoms in these animals. Infection in slaughter pigs and poultry can cause meat and egg contamination [39,40].

An infection with *Salmonella* generally occurs in three stages: the adhesion to the intestinal wall, the invasion of the gut wall and the dissemination to mesenteric lymph nodes and other organs. Via bacterial-mediated endocytosis, *Salmonella* invades the intestinal epithelial cells, after which the bacterium becomes enclosed within an intracellular phagosomal compartment (the *Salmonella*-containing vacuole (SCV)). After crossing the epithelial barrier, the bacterium is located predominantly in macrophages in the underlying tissue [39].

**Figure 2:**
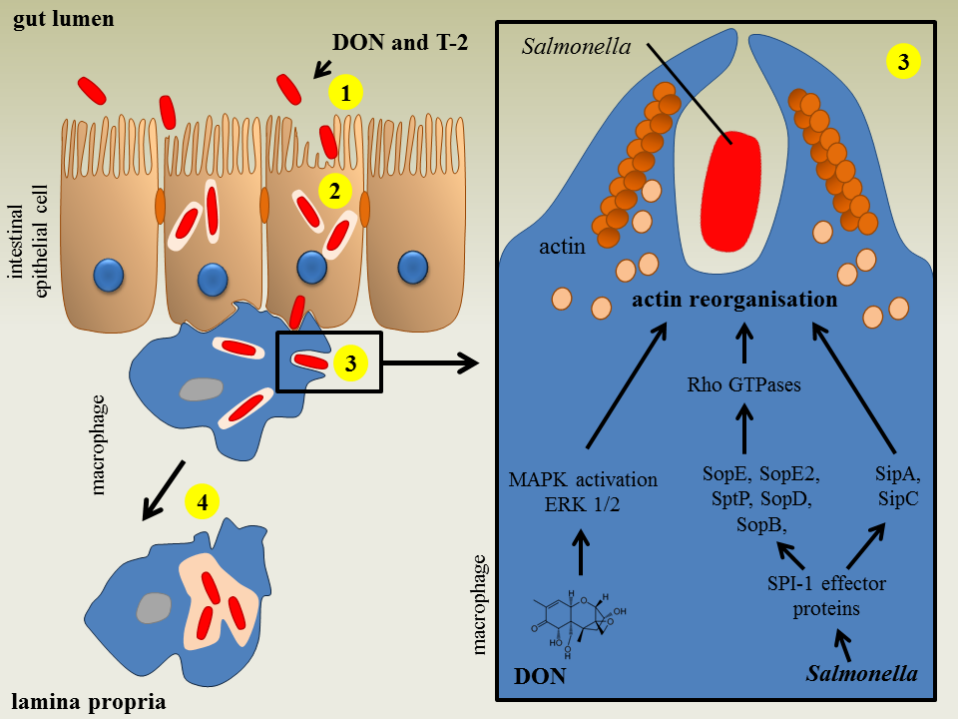
The impact of deoxynivalenol and T-2 toxin on a *Salmonella* Typhimurium infection in pigs. *In vitro*, deoxynivalenol (DON) and T-2 toxin (T-2) promote *Salmonella* invasion (1) and transepithelial passage (2) of IPEC-J2 cell layer. Subsequently, the bacterium can spread to the bloodstream using the host macrophage to establish the systemic infection. *In vitro*, DON and T-2 enhance *Salmonella* uptake (3) in porcine alveolar macrophages. The *Salmonella* invasion of macrophages coincides with membrane ruffling, caused by actin cytoskeletal changes. Activation of host Rho GTPases by the *Salmonella* pathogenicity island (SPI)-1 type 3 secretion system (T3SS) effector proteins SopB, SopE, SopE2 and SopD leads to actin cytoskeleton reorganization. After *Salmonella* internalization has occurred, the bacterium injects the effector protein SptP which promotes the inactivation of Rho GTPases. The bacterium can also modulate the actin dynamics of the host cell in a direct manner through the bacterial effector proteins SipA and SipC. The mycotoxin DON enhances the uptake of *Salmonella* in macrophages through activation of the mitogen-activated protein kinases (MAPK) extracellular signal-regulated kinases (ERK1/2) pathway, which induces actin reorganizations and membrane ruffles. DON and T-2 do not affect intracellular bacterial proliferation (4) (based on [41,44]).

Feeding pigs a *Fusarium* mycotoxin-contaminated diet influences the intestinal phase of the pathogenesis of *Salmonella* Typhimurium infections as illustrated in Figure 2. Non-cytotoxic concentrations of DON and T-2 enhance intestinal *Salmonella* invasion and increase the passage of *Salmonella* Typhimurium across the epithelium (Table A1) [28,41]. Chronic exposure of specific pathogen-free pigs to naturally fumonisin-contaminated feed had no impact on *Salmonella* Typhimurium translocation [42]. Once *Salmonella* has invaded the intestinal epithelium, the innate immune system is triggered and the porcine gut will start to produce several cytokines [28,43]. Both *Fusarium* mycotoxins and *Salmonella* affect the innate immune system. Vandenbroucke *et al*. [27] showed that low concentrations of DON could potentiate the early intestinal immune response induced by *Salmonella* Typhimurium infection. Co-exposure of the intestine to DON and *Salmonella* Typhimurium resulted in increased expression of several cytokines, for instance, those responsible for the stimulation of the inflammatory response (TNF-α) and T-lymphocyte stimulation (IL-12) (Table A2). The authors suggested that the enhanced intestinal inflammation could be due to a DON-induced stimulation of *Salmonella* Typhimurium invasion in and translocation across the intestinal epithelium [27].

*Fusarium* mycotoxins also affect the systemic part of the *Salmonella* Typhimurium infection in pigs. After the intestinal phase of the pathogenesis, *Salmonella* can spread to the bloodstream using the host macrophage to establish the systemic infection. However, in pigs the systemic part of *Salmonella* Typhimurium is poorly documented and colonization is mostly limited to the gastrointestinal tract [44]. After bacterial uptake by the macrophage, *Salmonella* can survive and even proliferate in this cell. Exposure of macrophages to non-cytotoxic concentrations of DON and T-2 promotes the uptake of *Salmonella* Typhimurium (Figure 2, Table A1). *Salmonella* entry in host cells involves a complex series of actin cytoskeletal changes. Macrophage invasion coincides with membrane ruffling, followed by bacterium uptake and formation of *Salmonella*-containing vacuole [41]. Vandenbroucke *et al*. [41] showed *in vitro* that DON enhances *Salmonella* Typhimurium engulfment, since low concentrations of DON modulate the cytoskeleton of macrophages through ERK1/2 F-actin reorganization resulting in an enhanced uptake of *Salmonella* Typhimurium in porcine alveolar macrophages (PAM) (Figure 2, Table A1). Non-cytotoxic concentrations of the *Fusarium* mycotoxins DON and T-2 did not affect the intracellular proliferation of *Salmonella* Typhimurium in porcine macrophages (Figure 2) [28,41].

In addition to the effects of *Fusarium* mycotoxins on the host susceptibility to a *Salmonella* Typhimurium infection, these mycotoxins also modulate the bacterial metabolism. Although no effect of DON or T-2 on the growth of *Salmonella* Typhimurium is detected, DON and T-2 modulate the *Salmonella* gene expression [28,41]. The enhanced inflammatory effect following exposure to DON is more likely a result of the toxic effect of the mycotoxin on the intestine than on the bacterium [27]. Only high concentrations of DON increase the bacterial expression of regulators of *Salmonella* pathogenicity island (SPI)-1 and SPI-2, respectively *hilA* and *ssrA*. SPI-1 consists of genes coding for bacterial secretion systems necessary for invasion, while SPI-2 genes encode essential intracellular replication mechanisms [41]. For T-2 the toxic effects on the bacterium itself are probably more pronounced than the host cell-mediated effects resulting in a reduced *in vivo* colonization in pigs. Low concentrations of T-2 cause a reduced motility of *Salmonella* and a general down regulation of genes involved in *Salmonella* metabolism, genes encoding ribosomal proteins and SPI-1 genes [28].

Only limited information is available concerning the interaction between *Fusarium* mycotoxins and *Salmonella* Typhimurium infection in other animals. The currently available publications mainly focus on the interaction of T-2 and the systemic phase of a *Salmonella* Typhimurium infection. In T-2-challenged broiler chickens and mice an increased level of *Salmonella* Typhimurium-related organ lesions or mortality was seen (Table A2) [45,46,47,48]. Infection of mice with *Salmonella* Typhimurium results in systemic infection and a disease similar to that seen in humans after infection with *Salmonella* Typhi [49]. Increased mortality might be explained partly by the synergistic effects of bacterial lipopolysaccharide (LPS) and T-2 during the late phase of murine salmonellosis [50]. In addition to *Salmonella* Typhimurium, DON reduces the resistance to oral infection with *Salmonella* Enteritidis in mice by promoting translocation of *Salmonella* to mesenteric lymph node (MLN), liver and spleen (Table A2) [51].

Mouse and pig models are important animal models to investigate the impact of mycotoxins, infectious diseases and their combination on animal health [52,53]. Infection of mice with *Salmonella* Typhimurium is an important host–pathogen interaction model to investigate typhoid fever in humans. Moderate to high concentrations of T-2 have shown to increase *Salmonella*-induced mortality [46,47,50]. The pig is very similar to humans in terms of anatomic and physiologic characteristics such as size, digestive physiology, kidney structure and function, pulmonary vascular bed structure, coronary artery distribution, respiratory rates, cardiovascular anatomy and physiology, and immune response, and has been used to study various intestinal pathogens, including *Salmonella* and *Escherichia coli* [53]. The interaction between mycotoxins and *Salmonella* Typhimurium studied in a porcine model of infection, gives us relevant information concerning the impact of this interaction on human intestinal inflammation and immune response [27].

In conclusion, the exact outcome of co-exposure to *Fusarium* mycotoxins and *Salmonella* Typhimurium is difficult to predict. Published data show an influence of mycotoxin exposure on the bacterium, the host cells and the host–pathogen interaction. Depending on the characteristics of the mycotoxin exposure, one of these effects will determine the outcome of the interaction between *Fusarium* mycotoxins and *Salmonella* Typhimurium.

### 3.2. Colibacillosis

*Escherichia coli* is a Gram-negative, non-sporulating rod-shaped bacterium of the family *Enterobacteriaceae*. Although this bacterium is considered to be a normal component of the intestinal microbiota, it is frequently associated with both intestinal and extra-intestinal infections in humans and animals. A certain number of these strains possess particular combinations of virulence factors which enables them to cause disease. Clinical syndromes resulting from infection with these pathotypes include enteric/diarrheal disease, urinary tract infections and sepsis/meningitis.

The pathogenesis of *E*. *coli* infections depends on the pathotype involved and may include colonizing the intestinal mucosa, evasion of host defenses, multiplication, and induction of host damage [54,55].

*Fusarium* mycotoxins may influence the pathogenesis of *E*. *coli* infections in different animal species by stimulating intestinal colonization and translocation and negatively affecting the immune response. Feeding a diet contaminated with a moderate level of FB1 to pigs enhanced intestinal colonization and translocation of a septicemic *E*. *coli* (SEPEC) strain from the intestine to the systemic compartment. FB1-treatment resulted in a higher bacterial translocation to the mesenteric lymph nodes and lungs, and to a lesser extent to liver and spleen (Table A2) [56]. It was shown *in vitro* that DON increased the translocation of SEPEC over the intestinal epithelial cell monolayer (IPEC-1) (Table A1) [14].

Mycotoxins increase the calf susceptibility to shiga toxin or verotoxin-producing *E*. *coli* (STEC)-associated hemorrhagic enteritis. Recently, Baines *et al*. [57] showed that exposing calves of less than one month old to the combination of aflatoxin and fumonisins promoted STEC-associated hemorrhagic enteritis (Table A2) [57].

Feeding a FB1-contaminated diet to pigs negatively affects the mucosal immune response against an infection with enterotoxigenic *E*. *coli* (ETEC). Devriendt *et al*. [58] showed a prolonged intestinal infection of *E*. *coli* in pigs administered fumonisins for 10 consecutive days and subsequently challenged with *E*. *coli* (F4^+^ ETEC) (Table A2). Antigen-presenting cells (APCs) have an important role in the mucosal immune system by connecting the innate and adaptive immune response, through uptake of antigen in lamina propria, maturation and migration to GALT, and interaction with T cells. FB1 negatively affected the function of intestinal APCs by a reduced up-regulation of the major histocompatibility complex class II (MHC-II), cluster of differentiation (CD) 80/6 and IL-12p40 cytokine gene expression [58]. This altered function of APCs could therefore influence the *E*. *coli*-induced adaptive immune response [58,59]. Additionally, moniliformin and FB1 delayed systemic *E*. *coli* (avian pathogenic *E*. *coli*, APEC) clearance in broilers and turkeys after intravenous administration (Table A2) [60,61].

The results of these studies may also be valid for human infections since the gastro-intestinal tract of pigs and humans are very similar [58]. Infant diarrhea caused by enteropathogenic *E*. *coli* (EPEC) is known to be of major concern in developing countries and, for instance, enterohemorrhagic *E*. *coli* (EHEC) infections are a major worldwide public health hazard.

### 3.3. Necrotic Enteritis in Broilers

Necrotic enteritis (NE) is a disease in broilers caused by *Clostridium perfringens*. This Gram-positive spore-forming bacterium occurs naturally in the environment, feed and gastrointestinal tract of chickens and other animals [62,63]. NE is a complex, multifactorial enteric disease with many known and unknown factors influencing its occurrence and the severity of the outbreaks. The best-known predisposing factor is mucosal damage caused by coccidial pathogens [64]. Only *C*. *perfringens* strains expressing the NetB toxin are capable of inducing NE in broilers [65]. *C*. *perfringens* is auxotrophic for several amino acids, thus availability of these amino acids would allow extensive bacterial proliferation [63].

The intake of DON-contaminated feed is a predisposing factor for the development of necrotic enteritis in broiler chickens due to the negative influence on the epithelial barrier, and to an increased intestinal nutrient availability for clostridial proliferation. Recently, we [66] showed in an experimental subclinical NE infection model that chickens fed a diet contaminated with DON for three weeks were more prone to develop NE lesions compared to chickens on a control diet (Table A2). The negative effects of DON on the small intestinal barrier can lead to an impaired nutrient digestion and leakage of plasma amino acids into the intestinal lumen, providing the necessary growth substrate for extensive proliferation of *C*. *perfringens* [66].

### 3.4. Edwardsiella ictaluri Infection in Catfish

*Edwardsiella ictaluri* is a Gram-negative bacterium of the *Enterobacteriaceae* family. Bacillary Necrosis of *Pangasianodon* (BNP) caused by *E*. *ictaluri* is the most frequently occurring infectious disease in catfish [67]. Besides the Vietnas the morphology and the barrier function of the intestinal layer [9], leading to increased translocation of different bacterial species including *Salmonella enterica* and *E*. *coli*, to the systemic compartment. The negative influence of these mycotoxins on the function of macrophages results in impaired phagocytosis of bacterial and fungal pathogens. However, also the adaptive immune response is targeted, demonstrated by the effect on gene expression of several cytokines, leading to an altered Th1 and Th2 response.

The economic impact of mycotoxins on animal production is generally considered to be mainly due to losses related to direct effects on animal health and trade losses related to grain rejection [91]. It is clear, however, that the indirect influence of myocotoxins on animal health, by enhancing infectious diseases, should also be taken into account. These effects, as reviewed here, occur even at low to moderate mycotoxin contamination levels of feed [8]. Some publications showed that these effects can even occur at contamination levels below the European guidance levels, suggesting that the legislation may not cover all deleterious health effects of mycotoxins.

*Fusarium* mycotoxins have various acute and chronic effects on humans [92]. DON could play a role in diseases such as inflammatory bowel disease (IBD) [20,93]. Taken into account conditions such as environmental, socio-economic and food production, it seems plausible that the risk for food-associated mycotoxin exposure is even higher in developing countries [94]. Besides the risk for acute mycotoxicosis in developing countries [95], results obtained in animals suggest that low to moderate concentrations of these mycotoxins could also influence human susceptibility to infectious diseases.

The effect of multi-mycotoxin contamination and of less well-known or emerging mycotoxins on the human or animal susceptibility to infectious diseases is rather unknown. Multi-mycotoxin contamination of feed is frequently occurring, raising the question on the impact on animal toxicity of this phenomenon [3]. Several *in vitro* and *in vivo* studies demonstrated an enhanced toxicity and more severe immune suppression compared to single mycotoxin contamination [96,97,98]. In addition, plant metabolites of mycotoxins may also be present in feed and are known as masked mycotoxins [99]. *Fusarium* fungi and infected plants may produce conjugated forms of, for instance, DON, such as 3-AcDON (3-acetylDON), 15-AcDON and DON-3G (DON-3-glucoside). Furthermore, mycotoxins can also be conjugated by certain food-processing techniques. These conjugated forms could have a direct toxic effect, or may be hydrolyzed to their precursor mycotoxin in the digestive tract of animals, resulting in higher exposure levels [100,101,102]. The influence of mycotoxin co-occurrence and masked mycotoxins on human and animal susceptibility to infectious diseases will be an important. Research question in the future.

Global warming and increasing world population of humans are further important issues. Climate changes may affect the global distribution of mycotoxigenic fungi and their mycotoxins [103,104], but also the distribution of infectious diseases [105]. Livestock farming will remain an important component of the global food supply in the future. Animal health, including the impact of mycotoxins and susceptibility to infectious diseases, will be important future topics to produce enough safe food for the entire human population.

In conclusion, *Fusarium* mycotoxins may alter the human and animal susceptibility to infectious diseases by affecting the intestinal health and the innate and adaptive immune system. Further research will be necessary to investigate the impact of mycotoxins on infectious diseases and to develop practical, economically justified, solutions to counteract mycotoxin contamination of feed and food, and its effects on human and animal health.

## Appendix

**Table A1:** Interaction between *Fusarium* mycotoxins and infectious diseases: *in vitro* approach.

Mycotoxin	Exposure dose	Exposure period	Cell line (host species)	Pathogen	Effect	Reference(s)
DON or T-2	>25 ng DON/mL or 5 ng T-2/mL; ≥0.75 µg DON/mL or ≥2.5 ng T-2/mL	24 h	undifferentiated IPEC^1^-J2; differentiated IPEC^1^-J2; (pig)	*Salmonella* Typhimurium	↑ invasion	[27, 28]
DON or T-2	0.5 µg DON/mL or ≥1.0 ng T-2/mL	24 h	differentiated IPEC^1^-J2 (pig)	*Salmonella* Typhimurium	↑ translocation	[27, 28]
DON or T-2	0.025 µg DON/mL or 1 ng T-2/mL	24 h	PAM^2^ (pig)	*Salmonella* Typhimurium	↑ invasion	[28, 41]
DON	5–50 µM (1.5–15 µg/mL)	48 h	IPEC^1^-J1 (pig)	*E*. *coli* (SEPEC)^3^	↑ translocation	[14]
T-2	0.001 µM	6 h	peritoneal macrophages (mouse)	*P*. *aeruginosa*^4^	↓ phagocytosis	[48]
T-2	0.01−0.05 µM	20 h	alveolar macrophages (rat)	*S*. *cerevisiae*^5^	↓ phagocytosis	[106]
T-2	0.1 µM	6 h	alveolar macrophages (rat)	*S*. *aureus*^6^	↓ phagocytosis	[106]
T-2	1–5 ng/mL; 2–5 ng/mL	24 h	HD-11 cell line^8^ (chicken)	*A*. *fumigatus*^7^	↓ phagocytosis; ↑ immune response^(A)^; ↑ germination	[80]

DON = deoxynivalenol; T-2=T-2 toxin; ^1^ IPEC = Intestinal Porcine Epithelial Cell; ^2^ PAM = porcine alveolar macrophage; ^3^ septicemic *Escherichia coli*; ^4^ *Pseudomonas aeruginosa*; ^5^ *Saccharomyces cerevisae*; ^6^ *Staphylococcus aureus*; ^7^ *Aspergillus fumigatus*; ^8^ chicken macrophages; ^(A)^ = increased gene expression of IL-1β, IL-6, CCLi1, CXCLi1, CXCLi2, IL-18 and IL-12β.

**Table A2:** The influence of *Fusarium* mycotoxins on infectious diseases in animals: *in vivo* approach.

Mycotoxin	Exposure dose	Exposure period	Animal species	Age	Pathogen	Effect: compared to negative control	Reference(s)
DON, 15-acetylDON, ZEN and fumonisins	6.5 mg DON, 0.44 mg 15-acetylDON, 0.59 mg ZEN and 0.37 mg fumonisins/kg feed	6 weeks	chicken (broiler)	1 day	*E*. *maxima* ^1^	↓ percentage of CD4^+^ and CD8^+^ T cells in jejunal mucosa	[35]
DON, 15-acetylDON and ZEN	3.8 mg DON and 0.3 mg 15-acetylDON and 0.2 mg ZEN/kg feed	10 weeks	chicken (broiler)	1 day	*E*. *acervulina* ^1^, *E*. *maxima* ^1^, *E*. *tenella* ^1^	↓ level of blood monocytes at end of challenge period; percentage of CD8^+^ T-cells not. Restored at end of recovery period; ↑ IFN-γ gene expression	[34]
DON, 15-acetylDON and ZEN	3.8 mg DON, 0.3 mg 15-acetylDON and 0.2 mg ZEN/kg feed	10 weeks	chicken (broiler)	1 day	*E*. *acervulina* ^1^, *E*. *maxima* ^1^, *E*. *tenella* ^1^	↓ intestinal recovery: duodenal villus height and apparent villus surface area	[36]
DON	1 µg/mL	6 h	pig	5 weeks	*Salmonella* Typhimurium	synergistic ↑ gene expression IL-12, TNF-α, IL-1β, IL-8, MCP-1 and IL-6	[27]
T-2	15 and 83 µg/kg feed	23 days	pig	3 weeks	*Salmonella* Typhimurium	↓ colonization of the cecum	[28]
FB1 and FB2	8.6 mg FB1 and 3.2 mg FB2/kg feed	9 weeks	pig	4 weeks	*Salmonella* Typhimurium	synergistic transient effect digestive microbiota balance	[42]
T-2	2 mg/kg BW	2 days	chicken (broiler)	1 day	*Salmonella* Typhimurium	↑ mortality	[45]
T-2	1 mg/kg BW	3 weeks	mouse	5–6 weeks	*Salmonella* Typhimurium	↑ mortality	[46]
T-2	1 mg/kg BW	10 days	mouse	5–6 weeks	*Salmonella* Typhimurium	↑ bacteria-related organ lesions	[47]
T-2	2 mg/kg BW	s.a.	mouse	-	*Salmonella* Typhimurium	↑ mortality	[48]
DON	1 mg/L drinking water	3 weeks	mouse	7 weeks	*Salmonella* Enteritidis	↑ translocation to mesenteric lymph node, liver and spleen	[51]
FB1	150 mg/kg feed	6 weeks	Japanese quail	1 day	*Salmonella* Gallinarum	↑ clinical signs and mortality; ↓ blood lymhocyte number	[107]
FB1	0.5 mg/kg BW	6 days	pig	3 weeks	*E*. *coli* (SEPEC) ^2^	↑ intestinal colonization; ↑ translocation to the mesenteric lymph node, lung, liver and spleen	[56]
FB1	1 mg/kg BW	10 days	pig	3–4 weeks	*E*. *coli* (ETEC) ^3^	intestinal infection prolonged; impaired function of intestinal antigen presenting cells	[58]
fumonisins and aflatoxin	^a^ 50–350 ng fumonisins /mL and 1–3 ng aflatoxin/mL	–	calf	<1 month	*E*. *coli* (STEC) ^4^	↑ susceptibility to hemorrhagic enteritis	[57]
moniliformin	75–100 mg/kg feed	3 weeks	chicken (broiler)	0 day	*E*. *coli* (APEC) ^5^	↓ bacterial clearance	[60]
moniliformin and FB1	100 mg moniliformin and 200 mg FB1/kg feed	3 weeks	turkey	0 day	*E*. *coli*^3^ (APEC) ^5^	↓ bacterial clearance	[61]
DON	4–5 mg/kg feed	3 weeks	chicken (broiler)	1 day	*C*. *perfringens* ^6^	↑ number of chickens with necrotic enteritis	[66]
DON	5–10 mg/kg feed	10 weeks	channel catfish	juvenile	*E*. *ictaluri* ^7^	↓ mortality	[71]
T-2	1–2 mg/kg	6 weeks	channel catfish	juvenile	*E*. *ictaluri* ^7^	↑ mortality	[70]
FB1, FB2 and FB3	20 mg FB1, 3.5 mg FB2 and 1.9 mg FB3/kg feed	42 days	pig	3 days	*M*. *hyopneumoniae* ^8^	↑ severity of the pathological changes	[76]
FB1	10 mg/kg feed	24 days	pig	3 days	*B*. *bronchiseptica* ^9^ *and P*. *multocida* ^10^ *(type D)*	↑ extent and severity of the pathological changes	[73]
FB1	0.5 mg/kg BW	7 days	pig	piglets	*P*. *multocida* ^10^ *(type A)*	↓ growth rate and ↑ coughing; ↑ total number of cells, number of macrophages and lymphocytes in BALF; ↑ gross pathological lesions and histopathological lesion of lungs	[74]
T-2	mg/mouse ≈ 3.3 mg/kg BW	20 days	mouse	adult	*M*. *tuberculosis* ^11^ (H37RvR-KM)	↑ bacterial count in spleen	[108]
T-2	0.1 mg/mouse ≈ 3.3 mg/kg BW	20 days	mouse	adult	*M*. *bovis* ^12^	↓ mouse survival time	[108]
T-2	0.5 mg/kg BW	21 days	rabbit	–	*A*. *fumigatus* ^13^	↓ phagocytosis by alveolar macrophages	[79]
T-2	2 mg/kg BW	s.a.	mouse	–	*P*. *aeruginosa* ^14^	↓ phagocytosis by peritoneal macrophages	[48]
DON	25 mg/kg BW	s.a.	mouse	7–10 weeks	reovirus (serotype 1)	↓ viral clearance and ↑ fecal shedding↓ Th1 response by ↓ IFN-γ gene expression↑ intestinal IgA and ↑ Th 2 response: by ↑ IL-4, IL-6 and IL-10 gene expression	[82]
T-2	1.75 mg/kg BW	s.a.	mouse	7–10 weeks	reovirus (serotype 1)	↓ viral clearance and ↑ fecal shedding; ↓ Th1 response by ↓ IFN-γ gene expression	[86]
FB1	12 mg/kg BW	18 days	pig	1 month	PRRSV^15^	↑ histopathological lesions of lungs	[89]

DON = deoxynivalenol; T-2 = T-2 toxin; ZEN = zearalenone; FB1 = fumonisin B1; FB2 = fumonisin B2; FB3 = fumonisin B3; BW = bodyweight; ^a^ mycotoxin level detected in the hemorrhaged mucosa; s.a. = single administration; ^1^ *Eimeria;* ^2^ septicemic *Escherichia coli*; ^3^ enterotoxigenic *Escherichia coli*; ^4^ shiga toxin producing *Escherichia coli*; ^5^ avian pathogenic *Escherichia coli*; ^6^ *Clostridium perfringens*; ^7^ *Edwardsiella ictaluri*; ^8^ *Mycoplasma hyopneumoniae*; ^9^ *Bordetella bronchiseptica*; ^10^ *Pasteurella multocida*; ^11^ *Mycobacterium tuberculosis*; ^12^ *Mycobacterium bovis*; ^13^ *Aspergillus fumigatus*; ^14^ *Pseudomonas aeroginosa*; ^15^ PRRSV = Porcine Reproductive and Respiratory Syndrome Virus.

**Table A3:** European Union limits for foodstuffs for human consumption, feed material and finished feed for animals adapted from the European Commission Regulation No 1881/2006 [109] and the European Commission Recommendations 2006/576/EC [110] and 2013/165/EU [111]

Mycotoxin	Foodstuffs for human consumption/finished animal feed	Maximum levels (µg/kg)
**DON**	unprocessed cereals other than durum wheat, oats and maize	1250
	unprocessed durum wheat and oats	1750
	unprocessed maize, with the exception of unprocessed maize intended to be processed by wet milling	1750
	cereals intended for direct human consumption, cereal flour, bran and germ as end product marketed for direct human consumption, with the exception of foodstuffs listed in ^(1)^.	750
	pasta (dry)	750
	bread (including small bakery wares), pastries, biscuits, cereal snacks and breakfast cereals	500
	^(1)^ processed cereal-based foods and baby foods for infants and young children	200
	*feed materials:*	
	cereals and cereal products with the exception of maize by-products	8000
	maize by-products	12,000
	*complementary and complete feedingstuffs:*	
	all animal species with the exception of ^(2)^	5000
	^(2)^ complementary and complete feedingstuffs for pigs	900
	^(2)^ complementary and complete feedingstuffs for calves (<4 months), lambs and kids	2000
**ZEN**	unprocessed cereals other than maize	100
	unprocessed maize with the exception of unprocessed maize intended to be processed by wet milling	350
	cereals intended for direct human consumption, cereal flour, bran and germ as end product marketed for direct human consumption, with the exception of foodstuffs listed in ^(2)^	75
	refined maize oil	400
	bread (including small bakery wares), pastries, biscuits, cereal snacks and breakfast cereals, excluding maize snacks and maize-based breakfast cereals	50
	^(2)^ maize intended for direct human consumption, maize-based snacks and maize-bases breakfast cereals	100
	^(2)^ processed cereal-based foods (excluding processed maize-based foods) and baby foods for infants and young children	20
	^(2)^ processed maize-based foods for infants and young children	20
	*feed materials:*	
	cereals and cereal products with the exception of maize by-products	2000
	maize by-products	3000
	*complementary and complete feedingstuffs:*	
	complementary and complete feedingstuffs for piglets and gilts (young sows)	100
	complementary and complete feedingstuffs for sows and fattening pigs complementary and complete feedingstuffs for calves, dairy cattle, sheep (including lamb) and goats (including kids)	250
	complementary and complete feedingstuffs for calves, dairy cattle, sheep (including lamb) and goats (including kids)	500
**Fumonisins (sum FB1 + FB2)**	unprocessed maize with the exception of unprocessed maize intended to be processed by wet milling	4000
	maize intended for direct human consumption, maize-based foods for direct human consumption, with the exception of foodstuffs listed in ^(3)^	1000
	^(3)^ maize-based breakfast cereals and maize-based snacks	800
	^(3)^ processed maize-based foods and baby foods for infants and young children	200
	*feed materials:*	
	maize and maize products	60,000
	*complementary and complete feedingstuffs:*	
	complementary and complete feedingstuffs for pigs, horses ( *Equidae*), rabbits and pet animals	5000
	complementary and complete feedingstuffs for fish	10,000
	complementary and complete feedingstuffs for poultry, calves (<4 months), lambs and kids	20,000
	complementary and complete feedingstuffs for adult ruminants (>4 months) and mink	50,000
**Sum T-2 and HT-2**	*unprocessed cereals:*	
	barley (including malting barley) and maize	200
	oats (with husk)	1000
	wheat, rye and other cereals	100
	*cereal grains for direct human consumption:*	
	oats	200
	maize	100
	other cereals	50
	*cereal products for human consumption:*	
	oat bran and flaked oats	200
	cereal bran except oat bran, oat milling products other than oat bran and flaked oats, and maize milling products	100
	other cereal milling products	50
	breakfast cereals including formed cereal flakes	75
	bread (including small bakery wares), pastries, biscuits, cereal snacks, pasta	25
	cereal-based foods for infants and young children	15
	*cereal products for feed:*	
	oat milling products (husks)	2000
	other cereal products	500
	*compound feed:*	
	compound feed, with the exception of feed for cats	250

(DON = deoxynivalenol, ZEN= zearalenone, T-2= T-2 toxin, HT-2= HT-2 toxin, FB1 = fumonisin B1, FB2 = fumonisin B2)
